# Analysis of Fall Risk Factors in an Aging Population Living in Long-Term Care Institutions in SPAIN: A Retrospective Cohort Study

**DOI:** 10.3390/ijerph17197234

**Published:** 2020-10-03

**Authors:** Lourdes Bujalance Díaz, María Jesús Casuso-Holgado, María Teresa Labajos-Manzanares, Francisco Javier Barón-López, Elena Pinero-Pinto, Rita Pilar Romero-Galisteo, Noelia Moreno-Morales

**Affiliations:** 1 Departament of Physiotherapy, DomusVi Remedios Center, Avda. Córdoba, 98, Aguilar de la Frontera, 14920 Córdoba, Spain; lourdes_fisioterapia@hotmail.com; 2 Department of Physiotherapy, Faculty of Nursing, Physiotherapy and Podiatry, University of Sevilla, C/Avicena s/n, 41009 Seville, Spain; epinero@us.es; 3 Department of Physiotherapy, Faculty of Health Sciences, University of Malaga, Arquitecto Francisco Peñalosa 3, Ampliación de Campus de Teatinos, 29071 Malaga, Spain; mtlabajos@uma.es (M.T.L.-M.); baron@uma.es (F.J.B.-L.); rpromero@uma.es (R.P.R.-G.); nmm@uma.es (N.M.-M.)

**Keywords:** aged, accidental falls, residential facilities, accident prevention

## Abstract

Falls in the elderly are associated with morbidity and mortality. Research about fall risk factors in Spanish care facilities is scarce. This study aimed to assess the prevalence of falls among residents living in long-term care Spanish institutions and to identify fall risk factors in this population. A nationwide retrospective cohort study was conducted in 113 centers. Persons over 70 years old who were living in a residential setting for at least 1 year were included. Simple and multiple regression analyses were conducted to estimate the associations between the main clinical variables registered in the databases and the presence of falls. A total of 2849 subjects were analyzed (mean age 85.21 years). The period prevalence of fallers in the last 12 months was 45.3%, with a proportion of recurrent fallers of 51.7%. The presence of falls was associated with lower Tinetti Scale scores (OR = 1.597, 95% CI: 1.280, 1.991; OR = 1.362, 95% CI: 1.134, 1.635), severe or moderate cognitive impairment (OR= 1.992, 95% CI: 1.472, 2.695; OR = 1.507, 95% CI: 1.231, 1.845, respectively), and polypharmacy (OR = 1.291, 95% CI: 1.039, 1.604). Fall prevention interventions should focus on the prevention of balance and cognitive deterioration and the improvement of these functions when possible. It should also focus on a periodical medication history revision aiming to avoid inappropriate prescriptions.

## 1. Introduction

Geriatric syndromes (GSs) are clinical conditions in elderly adults that do not fit into discrete disease categories and whose prevalence increases with age and physical frailty [1]. Although different conditions, such as incontinence, functional decline, or delirium, have been included under the heading of GSs, the concept is difficult to state [2,3]. An accepted definition of GSs is multifactorial health conditions that occur when the effects of impairments accumulate in multiple elderly systems [1].

Falls in the elderly are also reported as a GS. It is known that about 30–40% of persons above 65 years old suffer at least one fall a year [3,4,5,6], and 50% of them fall again after the first event [7].

Persons living in long-term care institutions have much higher rates of falls than the general population over 65 years [5,8]. Furthermore, falls among those in institutions also tend to result in more serious complications [9,10], such as significant effects on increased hospitalization rates, disability, increased mortality, and higher overall costs [3,11,12,13].

From a healthcare perspective, falls are considered to be an indicator of bad quality of care [14]. For this reason, fall risk factors have been widely researched in persons who live in nursing care or hospital settings, and they have been defined as gait instability, confusion, vertigo, cognitive impairment, urinary incontinence, history of falls, psychotropic and antihypertensive medications, and need for transfer assistance [1,4,15].

However, in residential and long-term care facilities, which are institutions along the continuum between living in one’s own private home in a community setting and living in a nursing home, research about fall risk factors is scarce. In a nationwide study in the United States, Towne et al. [16] observed that female residents needing assistance with activities of daily living were more likely to suffer a fall. On the contrary, smaller-sized facilities seem to be a protective factor. As the number of elderly adults in residential care facilities is expected to grow in the next decades, further research exploring multifactorial fall risk factors is recommended [16].

In Spain, the rate of people over 65 years old in 2020 is 19.58% [17], which supposes an increase of 2.78% of the population in the last decade. In 2011, a total of 270,000 people over 65 years old lived in long-term residential facilities, although this rate is expected to be higher nowadays. Research about fall incidence and fall risk factors in Spanish long-term care residents is scarce. Small sample sizes have been studied, and nationwide research is needed [13,18,19]. 

For this reason, the aim of this research was to assess the period prevalence of fallers among residents living in long-term care Spanish institutions in the last year and to identify fall risk factors in this population. This epidemiological data could be of interest for the implementation of successful fall prevention care programs.

## 2. Methods

This is a retrospective cohort study conducted in the main company of residential care institutions of Spain. A convenience sample was recruited from 113 long-term care institutions distributed across the different provinces of Spain.

Inclusion criteria were males and females 70 years of age and older living in a long-term care institution for at least 1 year. Exclusion criteria were the existence of physical sequelae related to a previous neurological disease, the existence of previous psychiatric disorder, complete blindness, or prescription of mechanical clamping. 

All the variables were registered by the health professionals involved in the care of the residents in each center as part of their periodical clinical evaluations. Thus, physicians, nursing, physical therapists, and psychology staff registered the information described above in the institutional database. As all the centers involved in this research belong to the same healthcare company, personnel at the different institutions are equally trained for evaluation and data registry.

### 2.1. Measures

#### 2.1.1. Outcome Variable 

Based on the event or not of at least a fall in the last year registered by nursing staff, subjects were classified into “fallers” and “non-fallers”. The period prevalence was calculated as the number of faller residents in the last year/the total sample studied (proportion of fallers). A fall was defined as proposed by the World Health Organization: “an event which results in a person coming to rest inadvertently on the ground or floor or other lower level” [20]. 

#### 2.1.2. Sociodemographic Factors

Age, gender, and time living in the care institution were registered [20,21,22,23].

#### 2.1.3. Balance and Walking Ability

It was assessed using the Tinetti Scale. It values balance and gait independently, with a maximum score of 28 points. A score < 19 points is considered as a high risk for falls. The assessment was performed by physical therapists [24,25].

#### 2.1.4. Cognitive Impairment

The level of cognitive impairment was assessed using the Mini-Mental State Examination (MMSE). This scale examines memory, orientation, and attention. The maximum total score is 30. The higher score is, the better the cognitive state of the patient. The assessment was performed by a psychologist or physician [26,27].

#### 2.1.5. Functional Independence

The level of functional independence was measured using the Barthel Index, which assesses the ability to execute 10 basic activities of daily living (BADLs). The score ranges from 0 to 100, and the maximum score indicates there will be no dependence. It is added at intervals of 5 points for each completed item. It was performed by occupational therapists [28,29]. 

#### 2.1.6. Medication

The number of drugs prescribed by physicians was collected to determine the condition of polypharmacy in the participants. Polypharmacy can be defined as “a high number of medications, but not inappropriate medications” [30] or as “the receipt of multiple medications” [31]. Although there are many definitions of polypharmacy in the literature, the most commonly reported definition of polypharmacy in the elderly population is based on the numerical definition of five or more medications daily [32]. Hence, this is the concept of polypharmacy stated in this research.

#### 2.1.7. Fall Prevention Interventions 

All activities that required mobility and that were supervised by a physical therapist were registered, such as performing strength exercises, stretching, aerobic exercise (such as walking, cycling, mobilizations, mechanotherapy or pole therapy), and postural and balance exercises. Physical exercise is expected to reduce the risk of falls [33] or the consequences of falls [34].

### 2.2. Procedure

First, ethical approval and permissions were obtained from the residential institution company. Afterwards, a statistician recruited all data from the electronic health record and anonymized it. These data were treated in accordance with legal regulations in force in Spain regarding personal data protection, especially Law 3/2018, of 5 July. This is a population-based retrospective cohort study based on medical records. The study was approved by the Review Board of the SARquavitae company on 26 April 2017. The project was identified by the title, but a code was not assigned to it. This research was also conducted in accordance with the Declaration of Helsinki.

Informed consent requirement was waived due to the study’s observational retrospective design on data that were obtained as part of routine clinical care. 

SARquavitae company has a restrictive policy in sharing data, which is a result of the General Data Protection Regulation.

Records were filtered in the database of each center based on the selection criteria and, in all cases, prior to the extraction of the final information. The records that were not updated in the last 6 months were also excluded. This procedure was supervised by the main researcher. 

### 2.3. Data Analysis

First, data were screened for any obvious errors, missing information, or duplications within the set. Descriptive statistics were obtained by measuring the central tendencies, and the rate of dispersion of the quantitative variables studied. Absolute and relative frequencies were also calculated for qualitative variables. A simple logistic regression analysis for each independent variable was performed to estimate the association with the presence of a fall in the past 12 months. Variables significant in bivariate analysis were subsequently included in a multivariate logistic regression model using forward conditional analysis. Confidence intervals were estimated at 95%. Statistical analysis was conducted using the SPSS program (v. 17.0).

## 3. Results

A total of 3028 records from 103 long-term care institutions were retrospectively screened, and 179 were excluded due to missing information. Thus, data from 2849 subjects (68.3% female) were finally analyzed in order to identify fall risk factors in a cohort of aged Spanish residents.

### 3.1. Sample Characteristics

The mean age of the sample was 85.21 (SD 6.6) years old, with a mean time of institutionalization of 3.38 years (SD 2.8). Differences by gender were observed; women were significantly older than men, with a shorter time of institutionalization. In general, women showed higher levels of physical and cognitive impairment. Significant differences between fallers and non-fallers were also found for all variables studied. Faller residents were older than non-fallers and showed lower levels of functional independence. Moreover, the number of medications was significantly higher in the cohort of fallers. 

Based on Tinetti Scale scores, 40.8% of the sample was at risk for falls, 59.8% of the residents had moderate to mild cognitive impairment, and 62.2% of the residents were moderately to slightly dependent. In all, 85.4% of the participants could be classified as being polymedicated, and 67% received physical therapy intervention. A more detailed description of the sample characteristics is reported in Table 1.

### 3.2. Associations with Past Year Fall and Fall Risk

A period prevalence of fallers in the last 12 months of 45.3% was observed with a proportion of recurrent fallers of 51.7% (people suffering more than one fall in the study period). An incidence of 3.1 new falls was also observed in this time. 

The association between the characteristics of participants and suffering at least a fall in the last 12 months (“faller”) was explored using logistic binary regression (Table 2). In the multivariate analysis, the presence of a fall was associated with lower Tinetti Scale scores (OR = 1.597, 95% CI: 1.280, 1.991; OR = 1.362, 95% CI: 1.134, 1.635), severe or moderate cognitive impairment (OR = 1.992, 95% CI: 1.472, 2.695; OR = 1.507, 95% CI: 1.231, 1.845, respectively), and polypharmacy (OR = 1.291, 95% CI: 1.039, 1.604). On the contrary, participants totally dependent or slightly dependent were less likely to suffer a fall (OR = 0.343, 95% CI: 0.193, 0.611; OR = 0.657, 95% CI: 0.487, 0.888, respectively). In the univariate analysis, the increased presence of a fall was also associated with being a woman (OR = 1.271, 95% CI: 1.083, 1.491) and being 85 years or older (OR = 1.317, 95% CI: 1.063, 1.632). Moreover, residents with a longer institutionalization period were less likely to suffer a fall (OR = 0.833, 95% CI: 0.703, 0.988). As we conducted a forward conditional analysis, we hypothesize that variables excluded from the multivariate analysis covariates with physical and cognitive impairment measures.

## 4. Discussion

The aim of this retrospective cohort study was to analyze the period prevalence of fallers in the last year among old persons living in long-term care institutions in Spain and to identify outcomes related to falls.

A total of 2849 participants from 113 long-term care institutions were analyzed. A fall prevalence of 45.3% was observed, with this rate being higher in women (71.2%) than in men (28.8%). The mean number of falls in the last year was 3.1.

Results from the multivariate logistic regression analysis reported that impaired balance and walking ability, impaired cognitive function, and polypharmacy were significant fall risk factors in the population of the study. The univariate analysis also reported that women and older people from both genders were more likely to suffer a fall; on the contrary, people with a longer stay in the center and not enrolled in physical therapy were less likely to suffer a fall. A possible explanation for the association between falls and physical therapy interventions is that the higher the physical impairment was, the more the prescription of physical therapy. Hence, we hypothesize that the variables excluded by multiple regression analysis covariate with having higher levels of physical and mental impairment. 

Our results are in agreement with Johanson et al. [23] who reported that community-dwelling women were at greater risk for falls compared with their male counterparts. Towne et al. [16] also observed a high prevalence of falls in female long-term care residents. In our case, this fact could be explained in part by a higher level of physical and cognitive deterioration being observed in women, but it could also be supported by the findings of Gale et al. [35], who reported that, in general, older women tend to fear pain and behave more carefully, making them less active than men. 

We also found that the length of stay in the center has an inverse relationship with falls, which could be explained by the fact that the elderly tend to lose mobility and functionality, as it can be observed for total dependency (OR: 0.343, 95% CI: 0.193, 0.611) [36]. As has been reported previously, fall rates are lower in frailer people [15]. 

The Tinetti Scale and Barthel Index assess physical skills, and both scales are considered to be good tools for fall risk prediction in accordance with Kaminska et al. [37] and Gronewold et al. [38]. However, our results support that the Tinetti Scale could be used as a better instrument to screen for fall risk in the elderly than the Barthel Index. 

Our results also support that cognitive status must be highly taken into account to assess the risk of falling. According to previous studies, the cognitive level seems to be important to determine the risk of falls [39]. However, our results are in disagreement with previous research in Spain, which concluded that there was no higher risk of falling observed among people with cognitive impairment or depression, although in this case, only community-dwelling older adults were studied [18]. These conflicting findings support that fall risk factors analysis may vary by setting and resident factors.

Another important finding is that polypharmacy can be considered a fall risk factor (OR: 1.291, 95% CI: 1.039, 1.604). This is congruent with previous research associating polymedication with an increased rate of falls [40,41]. It is also important to notice that the relationship between medication and falls in the elderly may not only be related to the numbers but also to the types of drugs [42,43,44,45]. Thus, future research should focus on this point.

This study has some limitations. First, although it was analyzed, a large sample was not randomly recruited. In fact, all subjects that met selection criteria were analyzed. Second, the geriatric evaluation was carried out by many different professionals from the 113 settings. Although health professionals are equally trained, this could have influenced the results. Third, the MMSE shows low sensitivity for patients with advanced dementia due to the “soil effect” (values lower than the minimum score are not possible to be identified, i.e., severe dementia) and is influenced by other sociodemographic variables, such as cultural level [26]. In future studies, it would be interesting to add other social variables such as cultural level or schooling, loneliness or depression, and to assess the existence of different comorbidities.

## 5. Conclusions

A high fall prevalence rate in the sample studied was observed, with women at more risk for falls than men. Balance and gait ability, cognitive status, and the number of medications can be considered important fall risk factors. Moreover, the Tinetti Scale and MMSE could be considered good tools for fall risk screening in the population of study. Based on our results, fall prevention interventions should focus on the prevention of balance and cognitive deterioration and the improvement of these functions when possible. It should also focus on a periodical medication history revision aiming to avoid inappropriate prescriptions.

## Figures and Tables

**Table 1:** Descriptive characteristics of the sample.

Descriptive Outcomes	Subgroups Mean (SD)/Proportions (%)			
	Total Sample (*n* = 2849)	Non-Faller (*n* = 1559, 54.7)	Faller ^1^ (*n* = 1290, 45.3)	*p*-Value
Gender				
Male	902 (31.7)	530 (34)	372 (28.8)	0.003
Female	1947 (68.3)	1029 (66)	918 (71.2)	
Age (Mean, SD)	85.21 (6.6)	84.76 (6.8)	85.76 (6.4)	<0.001
70–84 years	1189 (41.7)	688 (44.1)	501 (38.8)	
≥85 years	1660 (58.3)	871 (55.9)	789 (61.2)	
Institucionalization period	3.38 (2.8)	3.52 (3.1)	3.20 (2.6)	0.002
(Mean, SD)				
1–4 years	2124 (74.6)	1138 (73)	986 (76.4)	
≥5 years	725 (25.4)	421 (27)	304 (23.6)	
Tinetti Scale (Mean, SD)	21.26 (5.7)	21.68 (5.9)	20.75 (5.4)	<0.001
High risk of falls (1–18)	747 (26.2)	357 (22.9)	390 (30.2)	
Risk of falls (19–24)	1104 (38.8)	580 (37.2)	524 (40.6)	
No risk of falls (>24)	998 (35)	622 (39.9)	376 (29.1)	
MMSE Scale (Mean, SD)	19.16 (6.9)	20.04 (6.8)	18.10 (6.9)	<0.001
Severe impairment (<9)	280 (9.8)	123 (7.9)	157 (12.2)	
Moderate impairment (10–18)	950 (33.3)	467 (30)	483 (37.4)	
Mild impairment (19–23)	755 (26.5)	434 (27.8)	321 (24.9)	
Normal (24–30)	864 (30.3)	535 (34.3)	329 (25.5)	
Barthel Index (Mean, SD)	75.11 (22.2)	77.09 (22.6)	72.72 (21.6)	<0.001
Total dependency (0–20)	78 (2.7)	53 (3.4)	25 (1.9)	
Severe dependency (21–60)	631 (22.1)	282 (18.1)	349 (27.1)	
Moderate dependency (61–90)	1376 (48.3)	731 (46.9)	645 (50)	
Slight dependency (91–99)	396 (13.9)	272 (17.4)	124 (9.6)	
Independent (100)	368 (12.9)	221 (14.1)	147 (11.4)	
Number of drugs (Mean, SD)	8.07 (3.6)	7.93 (3.6)	8.25 (3.5)	0.019
1–4 drugs	415 (14.6)	246 (15.8)	169 (13.1)	
≥5 drugs	2434 (85.4)	1313 (84.2)	1121 (86.9)	
Physical therapy intervention	1908 (67)	1011 (64.8)	897 (69.5)	0.008
1–3 therapies	1161 (40.8)	606 (59.9)	555 (61.9)	
≥4 therapies	747 (26.2)	405 (40.1)	342 (38.1)	
Incidence of falls last 12 months (Mean, SD)			3.10 (3.1)	

^1^ Occurrence of at least a fall in the last year.

**Table 2:** Association between sample characteristics and suffering at least a fall in the last 12 months.

	Faller	
	Univariate OR (95% CI; *p*-Value)	Multivariate OR (95% CI; *p*-Value)
Gender		-
Male	Ref.	
Female	1.271 (1.083, 1.491; *p* = 0.003)	
Age		-
70–84 years	Ref.	
≥85 years	1.317 (1.063, 1.632; *p* = 0.012)	
Institucionalization period		-
1–4 years	Ref.	
≥5 years	0.833 (0.703, 0.988; *p* = 0.036)	
Tinetti Scale		
High risk of falls (1–18)	1.807 (1.491, 2.190; *p* < 0.000)	1.597 (1.280, 1.991; *p* < 0.001)
Risk of falls (19–24)	1.495 (1.256, 1.779; *p* < 0.000)	1.362 (1.134, 1.635; *p* = 0.001)
No risk of falls (> 24)	Ref.	Ref.
MMSE Scale		
Severe impairment (<9)	2.076 (1.580, 2.727; *p* < 0.000)	1.992 (1.472, 2.695; *p* < 0.001)
Moderate impairment (10–18)	1.682 (1.395, 2.028; *p* < 0.000)	1.507 (1.231, 1.845; *p* < 0.001)
Mild impairment (19–23)	1.203 (0.986, 1. 468; *p* = 0.069)	1.130 (0.921, 1.386; *p* = 0.243)
Normal (24–30)	Ref.	Ref.
Barthel Index		
Total dependency (0–20)	0.709 (0.422, 1.192; *p* = 0.709)	0.343 (0.193, 0.611; *p* < 0.001)
Severe dependency (21–60)	1.861 (1.433, 2.416; *p* < 0.000)	1.135 (0.839, 1.536; *p* = 0.410)
Moderate dependency (61–90)	1.327 (1.050, 1.676; *p* = 0.018)	1.037 (0.809, 1.330; *p* = 0.774)
Slight dependency (91–99)	0.685 (0.509, 0.923; *p* = 0.013)	0.657 (0.487, 0.888; *p* = 0.006)
Independent (100)	Ref.	Ref.
Number of drugs		
1–4 drugs	Ref.	Ref.
≥5 drugs	1.243 (1.006, 1.535; *p* = 0.044)	1.291 (1.039, 1.604; *p* = 0.021)
Physical therapy intervention		-
Yes	Ref.	
No	0.808 (0.690, 0.946; *p* = 0.008)	

*Note:* Ref.: reference.
